# Six Months of Piano Training in Healthy Elderly Stabilizes White Matter Microstructure in the Fornix, Compared to an Active Control Group

**DOI:** 10.3389/fnagi.2022.817889

**Published:** 2022-02-15

**Authors:** Kristin Jünemann, Damien Marie, Florian Worschech, Daniel S. Scholz, Frédéric Grouiller, Matthias Kliegel, Dimitri Van De Ville, Clara E. James, Tillmann H. C. Krüger, Eckart Altenmüller, Christopher Sinke

**Affiliations:** ^1^Division of Clinical Psychology & Sexual Medicine, Department of Psychiatry, Social Psychiatry and Psychotherapy, Hanover Medical School, Hanover, Germany; ^2^Center for Systems Neuroscience, Hanover, Germany; ^3^Geneva Musical Minds Lab, Geneva School of Health Sciences, University of Applied Sciences and Arts Western Switzerland, Geneva, Switzerland; ^4^Faculty of Psychology and Educational Sciences, University of Geneva, Geneva, Switzerland; ^5^Institute of Music Physiology and Musicians’ Medicine, Hanover University of Music, Drama and Media, Hanover, Germany; ^6^Swiss Center for Affective Sciences, University of Geneva, Geneva, Switzerland; ^7^Center for the Interdisciplinary Study of Gerontology and Vulnerability, University of Geneva, Geneva, Switzerland; ^8^Ecole Polytechnique Fédérale de Lausanne, Campus Biotech, Geneva, Switzerland; ^9^Faculty of Medicine, University of Geneva, Campus Biotech, Geneva, Switzerland

**Keywords:** healthy aging, fixel-based analysis, musical training, fornix, white matter, fiber density, episodic memory

## Abstract

While aging is characterized by neurodegeneration, musical training is associated with experience-driven brain plasticity and protection against age-related cognitive decline. However, evidence for the positive effects of musical training mostly comes from cross-sectional studies while randomized controlled trials with larger sample sizes are rare. The current study compares the influence of six months of piano training with music listening/musical culture lessons in 121 musically naïve healthy elderly individuals with regard to white matter properties using fixel-based analysis. Analyses revealed a significant fiber density decline in the music listening/musical culture group (but not in the piano group), after six months, in the fornix, which is a white matter tract that naturally declines with age. In addition, these changes in fiber density positively correlated to episodic memory task performances and the amount of weekly piano training. These findings not only provide further evidence for the involvement of the fornix in episodic memory encoding but also more importantly show that learning to play the piano at an advanced age may stabilize white matter microstructure of the fornix.

## Introduction

The World Health Organization (WHO) appointed the years 2020–2030 as the decade of healthy aging and considers a person’s intrinsic capacity as one of the main components of healthy aging. This includes physical movement, vision, hearing, vitality, cognition, and psychological well-being (World Health Organization, 2020).

In recent years, evidence accumulated that playing a musical instrument represents an activity to promote healthy aging as it targets most of the intrinsic capacities identified by the WHO. For example, Verghese et al. (2003) showed that frequently playing an instrument reduced the risk of developing dementia. While this study demonstrated effects for people who were already proficient in playing an instrument, a few other studies have investigated musically naïve older individuals who started learning to play an instrument later in life. Some initial studies showed improved executive functioning and working memory (Bugos et al., 2007), improved executive functioning and an increased quality of life (Seinfeld et al., 2013), and a possible effect for improved verbal and visual memory (but no increased executive functioning) (Degé and Kerkovius, 2018) after up to six months of instrumental training. The discrepancies between studies could be due to the relatively small sample sizes of 24–29 subjects and differences in psychometric testing. As these studies did not use any neuroimaging methods, the underlying neural mechanisms of these changes could not be probed. However, since accumulative evidence shows that, in the adult brain, learning a new skill (e.g., dancing, music training or juggling) can lead to experience-dependent structural changes, it is probable that this is also the case in healthy elderly individuals when starting to learn an instrument later in life (Boyke et al., 2008; May, 2011).

Since making music is a popular tool for studying brain plasticity, an extensive amount of literature is available on how brains of musicians differ from the brains of non-musicians. One important aspect is how lifelong music making affects the communication between different brain regions and therefore structural connectivity (white matter) in the brain. This has mostly been investigated using diffusion tensor imaging (DTI), a method that predominantly uses fractional anisotropy (FA) as a measure, with lower FA values often being interpreted as decreased white matter fiber tract integrity (Alexander et al., 2007).

Studies found higher FA values in the right internal capsule, which is a part of the corticospinal tract (Bengtsson et al., 2005), and the arcuate fasciculus (Halwani et al., 2011), in musicians when compared to non-musicians. Additionally, practice time during childhood has been shown to positively correlate with FA values in the internal capsule and the corpus callosum (Bengtsson et al., 2005). However, other studies found lower FA values in the internal capsule (Schmithorst and Wilke, 2002) and corticospinal tract (Imfeld et al., 2009). These differences in findings make it difficult to draw strong conclusions about how music making exactly influences structural connectivity in the brain.

One important factor leading to these inconsistent findings might again be the relatively small sample sizes (*N* = 5–18, per group), which emphasizes the need for long-term randomized controlled trials (RCTs) with larger sample sizes. Another problematic factor might be the use of the diffusion tensor model, which can be unreliable and difficult to interpret (Mito et al., 2018). The main issue is that classical DTI analysis estimates one value per voxel and does, therefore, not distinguish different fiber populations within a specific voxel, although findings suggest that these crossing fibers are present in 60–90% of white matter voxels (Jeurissen et al., 2013). A relatively new technique called fixel-based analysis (FBA) uses constrained spherical deconvolution (CSD; Tournier et al., 2007), which allows to model different fiber populations within the same voxel, providing biologically meaningful markers for these individual fiber populations, the so called “fixels” (Raffelt et al., 2015, 2017b). FBA enables to study microscopic as well as macroscopic white matter changes in vivo. Microstructural changes can be investigated using fiber density (FD), reflecting intra-axonal fiber bundle volume, while fiber-bundle cross-section (FC) represents fiber bundle width and can therefore reflect macrostructural changes (Raffelt et al., 2017b). In addition, a combined measure exists, which is called fiber density and cross-section (FDC).

Several studies have used FBA to show how aging affects white matter connections in the brain. A cross-sectional study, for example, revealed a variety of age-related changes, including FC and FDC reductions in the corticospinal tract and a negative correlation between age and FD in the corpus callosum (Choy et al., 2020). Another study showed age related FD reductions in the fornix (Radhakrishnan et al., 2020), which is a main output tract of the hippocampus and mainly involved in episodic memory (Sexton et al., 2010). Furthermore, Koops et al. (2021) found a negative correlation between age and FD in the left and right acoustic radiations, which connect the thalamus to the auditory cortices, conveying auditory information. In this tract, FBA is especially helpful, as acoustic radiation fibers cross many major white matter fiber tracts in the internal capsule on the way from the thalamus to the cortex. Therefore, this tract is often overlooked when using classical DTI approaches (Maffei et al., 2019).

Taking both music and aging literature into consideration, learning to play an instrument might be a way to counteract or slow down these age-related white matter changes. This study aims to test this hypothesis with a long-term RCT, comparing two groups of musically naïve healthy elderly individuals, one group learning to play the piano (PP, the intervention group), and the other group engaging in music listening/musical culture lessons (MC, the active control group). The active control group has the advantage that it allows to assess the specific benefits of auditory-sensory-motor integration on neuroplasticity induced by piano learning and differentiate it from bare music listening, enjoyment and social interaction effects.

We took advantage of FBA to investigate white matter microscopic and macroscopic changes induced by musical training in our study population. We decided to focus on specific tracts of interest (TOIs) in the brain that could be influenced by the intervention. Based on the above-mentioned literature, we focused on eight TOIs: the corpus callosum, left and right corticospinal tract, left and right arcuate fasciculus, fornix, and left and right acoustic radiation. We anticipated less decline or even an increase in white matter microstructure and/or macrostructure through piano lessons in these TOIs. Additionally, we sought to correlate neuronal changes and behavioral changes and determine their relationship to training intensity.

## Materials and Methods

The study protocol was approved by the local ethics committees. All participants gave written informed consent before participation and all experiments were compliant with the Declaration of Helsinki. The study protocol was additionally registered on ClinicalTrials.gov (number 81185).

### Participants and Study Design

A total of 155 musically naïve subjects (92 females) were recruited as part of this longitudinal study in Hannover (Germany, 92 participants) and Geneva (Switzerland, 63 participants). Participants were 62–78 years of age (mean age = 69.7 years, std = 3.5), right-handed (Oldfield, 1971), retired and did not rely on hearing aids. Participants had not receive more than 6 months of formal musical training outside the school curriculum during their life prior to this study. People with neurological, psychological, severe physical health or cognitive impairment were not included in the study. Cognitive functioning was assessed using the Cognitive Telephone Screening Instrument (COGTEL; Ihle et al., 2017; Kliegel et al., 2007). This cognitive test, developed specifically for older adults, provides a global measure of cognition, based on several memory and executive function subtests. A detailed list of inclusion and exclusion criteria can be found in James et al. (2020).

Participants were randomly assigned to the PP group or the MC group taking age, gender, education level and COGTEL score into account to achieve equal distributions of these factors across groups. Participants were informed about their group assignment after completion of the baseline testing and retested after six months of lessons. During baseline testing experimenters were masked for the group allocation but afterwards were unmasked.

In all, 136 participants completed diffusion-weighted imaging at both time points. Ten participants dropped out of the study, because of time (3), health (2) and/or family issues (1), lack of interest (3), or stress (1) and nine participants were not comfortable with MRI scanning. Of these 136 participants, three participants had to be excluded because of artifacts in the T1 or diffusion images, one participant was excluded because of too much atrophy and myelin degeneration, three participants moved excessively during diffusion measurements and for eight participants the registration from subject to template space did not work properly, therefore they could not be used for analyses. This resulted in a total of 121 participants who were included in the final analysis (62 in the MC group and 59 in the PP group).

### Interventions

Participants received 60-min lessons once a week for 6 months and were additionally instructed to spend at least 30 min on homework each day. Professional musicians who held at least a bachelor’s degree and were supervised by music education and piano pedagogy specialists taught both intervention groups. Here only a brief description of the interventions is given. A more detailed version can be found in James et al. (2020). Further, the supplementary material of Worschech et al. (2021) includes a detailed description of the intervention curriculum.

#### Piano Practice

PP students were instructed in dyads. All participants were equipped with a Yamaha P-45 B keyboard and a height adaptable piano stool for at home practicing. Lessons included clapping and singing to familiarize the students with musical rhythm and music score reading. Playing with both hands (separately at first), reading music, and playing different styles of expressive music constituted the courses. Homework consisted of repeating pieces learned during class, learning new pieces, and improvising.

#### Music Listening/Musical Culture

MC lessons were given in small groups of 4–6 participants. Different musical styles were introduced ranging from classical to jazz and pop. Additionally, lessons included learning about different musical instruments and composers. Active listening, experiencing, and appreciating music was encouraged by the instructors. Any music-related movement, including clapping and singing, was omitted during the lessons. As homework, the participants actively listened to different musical pieces, read texts and prepared presentations.

### Image Acquisition and Preprocessing

Diffusion-weighted images were acquired on 3.0 T Siemens MRI scanners (Hannover: Magnetom Skyra, Geneva: Magnetom Tim Trio; Siemens, Erlangen, Germany) using 32-channel head coils. Both study sites used the same scanning parameters: voxel size = 1.5 mm isotropic, FoV = 222 mm × 222 mm × 126 mm, multiband acceleration factor = 3, repetition time (TR) = 5701 ms, echo time (TE) = 113.4 ms, and 84 slices. Sixty images were acquired with b = 1,500 s/mm^2^ and six interleaved images without diffusion weighting (b = 0 s/mm^2^) with an anterior to posterior phase encoding direction. One additional non-diffusion-weighted image was recorded in the reverse phase encoding direction to enable susceptibility-induced distortion correction (Andersson et al., 2003). In addition a high-resolution T1-weighted structural image was acquired, using the following MP2RAGE sequence (Marques et al., 2010): voxel size = 1 mm isotropic, FoV = 256 x 240 x 176 mm, TR = 5000 ms, TE = 2.98 ms, flip-angle 1 = 4°, flip-angle 2 = 5°, and 176 slices. T1 images were used to create a brain mask (using FreeSurfers recon-all command) for each subject for subsequent analyses.

Preprocessing of the diffusion images included denoising (Veraart et al., 2016) and Gibbs ringing removal (Kellner et al., 2016) of the data in MRtrix3 (Tournier et al., 2019) before correcting for susceptibility-induced distortions (Andersson et al., 2003), subject motion and eddy currents (Andersson and Sotiropoulos, 2016) using FSL (Smith et al., 2004). Afterward, the data were corrected for bias fields using the ANTs package (Tustison et al., 2010; Avants et al., 2011). To account for image intensity differences between both sites, images were scaled into a common intensity range using the mrhistmatch scale command, provided in MRtrix3. Preprocessed data was visually inspected to ensure that all previous steps had worked.

### Fixel-Based Analysis

If not otherwise noted, the recommended FBA processing pipeline (Raffelt et al., 2017b) was used. The following steps were performed using MRtrix3 and MRtrix3 Tissue,^1^ a fork of MRtrix3 (Tournier et al., 2019).

In a first step, response functions were computed for gray matter, white matter, and cerebrospinal fluid for each individual (Dhollander et al., 2016, 2019) and averaged over all participants to obtain a unique response function for each tissue type. The group averaged response functions were used to compute fiber orientation distributions (FODs) using Single-Shell, 3-Tissue Constrained Spherical Deconvolution (SS3T-CSD; Dhollander and Connelly, 2016), followed by joint bias field correction and intensity normalization (Raffelt et al., 2017a). Twenty participants (10 from the PP group, 10 from the MC group, half women, and half men) from each time point were randomly selected to generate an unbiased study-specific FOD template (population template) to which all the FODs of participants were non-linearly registered (Raffelt et al., 2011, 2012) and segmented to get fiber-specific fixels within each voxel. As described by Raffelt et al. (2017b), FBA metrics (FD, FC, and FDC) were calculated for each fixel. Further FC values were transformed into log space as recommended (Dhollander et al., 2021). In the last step, probabilistic tractography was used to generate a whole-brain tractogram on the population template. Twenty million streamlines were initially generated and then filtered to 2 million streamlines using spherical-deconvolution informed filtering of tractograms (Smith et al., 2013) to reduce reconstruction bias in the tractogram.

### TOI Analysis

Fixel-based analysis parameters were examined in eight TOIs: the corpus callosum, fornix, left and right acoustic radiation, left and right corticospinal tract, and left and right arcuate fasciculus. All masks, except for the arcuate fasciculi, were taken from the JHU white matter tractography atlas (Mori et al., 2005; Wakana et al., 2007; Hua et al., 2008) implemented in FSL. As the arcuate fasciculi are not defined in this atlas, inclusion masks (inferior frontal, inferior parietal, and superior temporal gyri) were derived from the Harvard-Oxford cortical and subcortical structural atlases (Frazier et al., 2005; Desikan et al., 2006; Makris et al., 2006; Goldstein et al., 2007). All masks were binarized and non-linearly transformed to the population template using MRtrix3. Tractography was then performed in template space using the probabilistic iFOD2 algorithm (Tournier et al., 2010). The generated TOIs were converted to fixel masks for statistical analyses.

### Behavioral Tests

We decided to restrict our analysis on hypothesis-driven relevant behavioral tests in case of significant FBA results for each TOI beforehand. Therefore, only these tests are explained in detail below. For a full list of administered tests in the study, please see James et al. (2020).

#### Rey Auditory Verbal Learning Test

Participants undertook the Rey Auditory Verbal Learning Test (RAVLT) to assess episodic (verbal) memory. This can be measured with the RAVLT delayed recall condition (RAVLT-DelayedR). First the experimenter read aloud a list of 15 unrelated words and asked the participants afterward to repeat these words. They repeated this procedure four times. After a delay of approximately 30 min (in the meantime the experimenter administered other tests) the experimenter asked the participants to repeat as many words from the list as they could remember without oral presentation beforehand. The number of the correctly recalled words is the RAVLT-DelayedR score. For measurements at baseline and 6 months different word lists were used to minimize the retest effect. In Hannover the German version (Helmstaedter et al., 2001) and in Geneva the French version (Rey, 1964) was used.

#### MIDI-Based Scale Analysis

To measure whether the piano training led to measurable behavioral improvements we used a MIDI-based Scale Analysis (MSA; Jabusch et al., 2004). The procedure was adapted to factor in that the participants had no piano experience before the start of the intervention. The experimenter instructed the participants to play sequences of 15 five-tone range scales (C-G) in both playing directions with the right hand. They were instructed to use the following fingering: 1-2-3-4-5-4-3-2-1-2-3-4-5-4-3-2-1, where the numbers correspond to the fingers (1 = thumb, 5 = little finger). The playing tempo was paced by a metronome with a desired inter-onset interval (IOI, time between the onsets of two subsequent notes) of 790 ms. The outcome measure was the mean standard deviation of the IOI, calculated for all played scales. This measure has previously been shown to be an indicator of pianistic expertise (Jabusch et al., 2009).

### Statistical Analysis

MRtrix3 was used for all FBAs. As longitudinal group comparisons are difficult to model in a General Linear Model (GLM) in MRtrix3, difference scores between the two time points (6 months—baseline) for each participant and FBA metrics (FD, (log)FC, and FDC) were calculated; all subsequent analyses were performed on these difference images. Separate analyses were run for each TOI mask for each FBA metric to assess group differences between the MC and PP groups. The data was entered into a GLM, using age (at start of the intervention, demeaned), gender, site and time between scans (in weeks, demeaned) as covariates. Connectivity-based fixel enhancement (CFE; Raffelt et al., 2015) was run with 5,000 permutations, which returned family wise error (FWE) corrected p values for each fixel in the TOI mask images. To account for multiple testing, Bonferroni correction was performed for the eight TOIs, considering *p* < 0.00625 as the significant threshold.

All other statistical analyses were performed in IBM SPSS Statistics version 26 (IBM Corp., Armonk, NY, United States) and R (R Core Team, 2021). As difference scores were fed into the GLM, the outcome does not quantify where the difference after 6 months of training stems from. This model only tests whether there is a difference between the two groups over time. Therefore, the mean value over significant fixels (after Bonferroni correction) were extracted from each participant and a one-sample *t*-test to check whether each group significantly differed from 0 (which would quantify change over time) was applied.

In a last step, it was analyzed whether extracted fixel results could be related to behavioral data. Difference scores for the behavioral measures were calculated (6 months—baseline) before being entered into a partial correlation, controlling for age (at the start of the intervention), site, and gender. Non-parametric partial correlation coefficients (using Spearman) were calculated with the R package ppcor (Kim, 2015), as site and gender are nominal variables and not suitable for parametric analyses. Bonferroni was again used to correct for multiple comparisons, with a significance threshold of *p* < 0.025. The RAVLT-DelayedR was analyzed with an independent two-sample t-test between difference scores and the MSA with a repeated measures design.

## Results

### Demographic Data

There were no significant group differences between the MC and PP groups in the following parameters: age (at start of the intervention), education level, gender ratio, COGTEL score, and for weekly amount of homework. See Table 1 for group means, statistics and *p*-values.

**TABLE 1:** Demographic data and group comparisons (*N* = 121).

	Mean (std)		Group comparison		
	PP	MC	statistic	*p*	95% CI
Age	69.49 (3.22)	69.42 (3.79)	*t* = 0.11	0.91	−1.19, 1.33
Males/ Females	27/32	25/37	*χ^2^* = 0.37	0.55	
Education	3.93 (1.39)	4.05 (1.34)	*χ^ 2^* = 2.42	0.79	
COGTEL score	30.66 (6.72)	32.23 (7.48)	*t* = −1.21	0.23	−4.13, 0.99
Weekly homework (min)	269.58 (142.49)	232.26 (121.44)	*t* = 1.55	0.12	−9.42, 85.75
RAVLT-DelayedR	0.153	0.016	*t* = 0.323	0.75	−0.699, 0.972

*Education was measured in different levels (1 = primary school, 2 = middle school, 3 = high school, 4 = Bachelor’s degree, 5 = Master’s degree, 6 = doctorate degree). COGTEL, Cognitive Telephone Screening Instrument; RAVLT-DelayedR, Rey Auditory Verbal Learning Test (delayed recall); MC, music listening/musical culture group; PP, piano playing group; CI, confidence Interval. Group differences were calculated using a two-sample independent t-test for age, COGTEL score, weekly homework, and RAVLT-DelayedR score difference and a chi-squared test for males/females and education.*

### Group Differences in TOIs

We investigated differences between the MC and PP groups over 6 months in eight TOIs for the following three FBA measures: FD, FC, and FDC.

**FIGURE 1:**
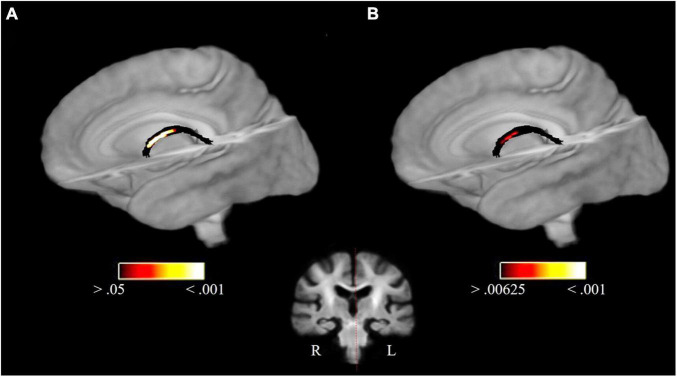
FD group differences in the fornix for PP > MC. Significant fixels in the fornix at p_FWE_ < 0.05 **(A)** and significant fixels after Bonferroni correction (p_FWE_ < 0.00625) **(B)** overlaid on the population template. The coronal section shows the location of the findings.

FBA revealed significant FD differences for PP > MC in the body of the fornix (*p*_FWE_ = 0.0052, *t*(120) = 3.51, *d* = 0.64, fixel count = 6, for *p*_FWE_ < 0.00625; *t*(120) = 3.01, *d* = 0.55, fixel count = 20, for *p*_FWE_ < 0.05). This result remained significant after Bonferroni correction. Figure 1 shows a visualization of this result before and after Bonferroni correction. No other significant group difference occurred for FD. In addition, the analysis revealed FWE-corrected significant (log)FC differences for PP > MC in the left acoustic radiation (*p*_FWE_ = 0.036, *t*(120) = 2.61, *d* = 0.48, fixel count = 20, for *p*_FWE_ < 0.05) and fornix (*p*_FWE_ = 0.024, *t*(120) = 3.09, *d* = 0.56, fixel count = 10, for *p*_FWE_ < 0.05), as well as for FDC in the splenium of the corpus callosum (*p*_FWE_ = 0.039, *t*(120) = 3.47, *d* = 0.63, fixel count = 13, for *p*_FWE_ < 0.05) and fornix (*p*_FWE_ = 0.0066, *t*(120) = 2.93, *d* = 0.053, fixel count = 24, for *p*_FWE_ < 0.05). However, all of these other above-mentioned results do not survive Bonferroni correction for the number of TOIs. No significant MC > PP group effects occurred.

### Group Differences in the Fornix

To investigate the nature of the FD group difference in the fornix, significant fixels (Bonferroni corrected) were extracted and a one sample t-test for both intervention groups was performed.

*Post hoc* tests revealed that the mean FD difference score in the MC group (mean = −0.0231, std = 0.039) was significantly different from 0 [*t*(61) = −4.584, *p* < 0.001, *d* = −0.582, 95% CI = −0.033, −0.013], indicating a decline in FD over 6 months. Whereas the difference score in the PP group (mean = 0.0047, std = 0.038) was not significantly different from 0, indicating no change over time [*t*(58) = 0.947, *p* = 0.347, 95% CI = −0.005, 0.015].

**FIGURE 2:**
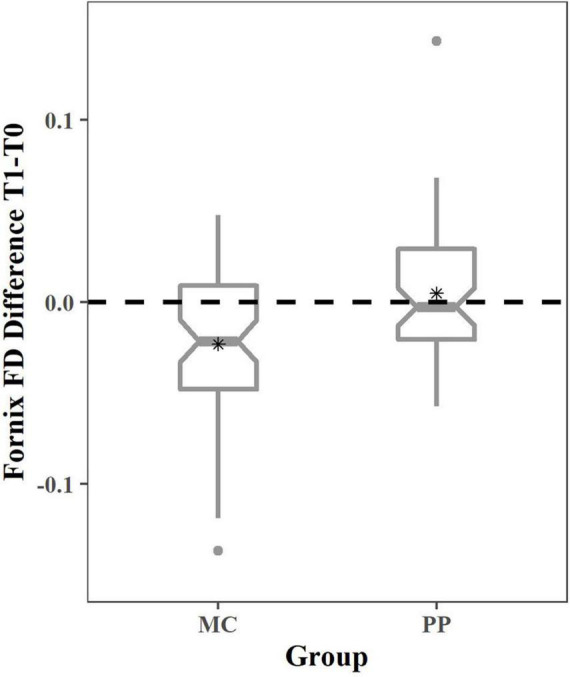
Fiber density change in MC and PP in the fornix. Boxplots depicting the change in FD for the MC and PP group. The dotted line represents no change over time and asterisks (*) depict the group mean. One-sample *t*-tests revealed no significant change over time in PP and significant FD decrease in MC. T0, baseline measurement; T1, 6 months measurement; MC, music listening/musical culture; PP, piano practice.

Therefore, the group difference after 6 months seems to arise because of an FD decrease in the MC group and absence of microstructural changes in the PP group (Figure 2).

### Change of Fornix Microstructure in Relation to Episodic Memory

As the fornix plays a role in episodic memory (Sexton et al., 2010; Metzler-Baddeley et al., 2011), we investigated the results of the RAVLT-DelayedR. In a cross-sectional investigation, the RAVLT-DelayedR was linked to fornix FD (Radhakrishnan et al., 2020). We therefore wanted to investigate whether this association could be confirmed in a longitudinal design. An independent two sample *t*-test revealed no group differences between the two groups for the RAVLT-DelayedR difference scores (Table 1).

**FIGURE 3:**
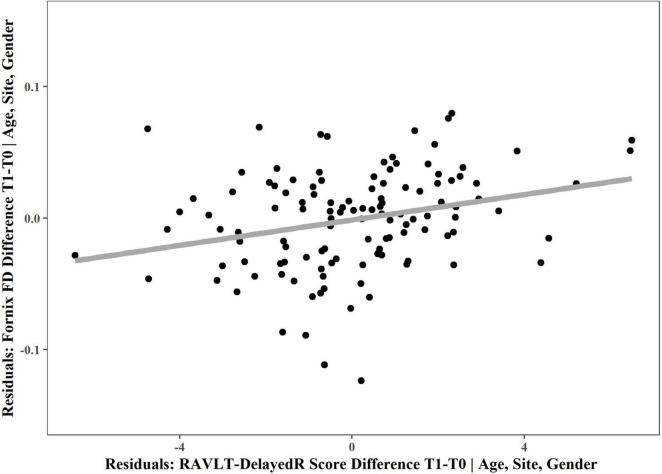
Non-parametric partial correlation between fornix FD and RAVLT-DelayedR changes. Non-parametric partial correlation, controlling for age (at the start of the intervention), site and gender revealed a significant correlation between fornix FD change and change in RAVLT-DelayedR performance (measuring episodic (long-term) verbal memory). For visualization, the residuals of multiple regression are plotted and a linear regression line was chosen to depict the positive correlation. T0, baseline measurement; T1, 6 months measurement.

Therefore, we decided to merge both intervention groups together to examine whether the fornix FD changes related to RAVLT-DelayedR scores using a non-parametric partial correlation, which revealed a positive correlation between the fornix FD and RAVLT-DelayedR changes (*r* = 0.237, *p* = 0.0097; Figure 3). This indicates a weak to moderate relationship between changes in fornix microstructure and episodic (long term) verbal memory across both groups.

### Change of Fornix Microstructure in Relation to Piano Training Intensity

**FIGURE 4:**
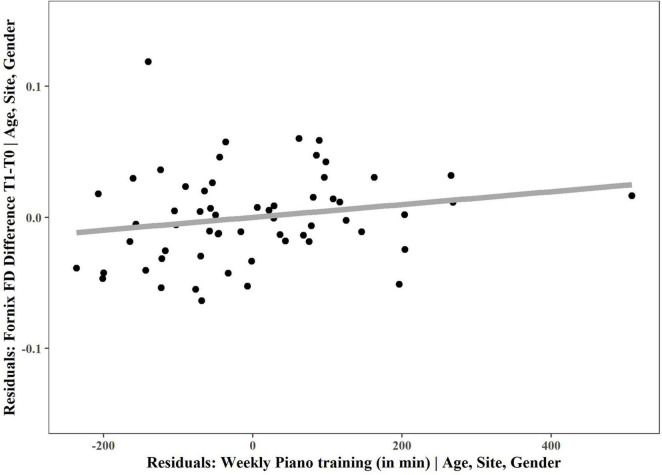
Non-parametric partial correlation between fornix FD and weekly piano training. Non-parametric partial correlation, controlling for age (at the start of the intervention), site and gender revealed a significant correlation between fornix FD change and weekly piano training. For visualization, the residuals of multiple regression are plotted and a linear regression line was chosen to depict the positive correlation. T0, baseline measurement; T1, 6 months measurement.

In a next step, we checked whether the FD change in the fornix correlated with the amount of weekly piano training at home, which was confirmed by a significant positive non-parametric partial correlation between weekly piano training and change in fornix FD (*r* = 0.326, *p* = 0.014; Figure 4).

### Piano Training Progress

**FIGURE 5:**
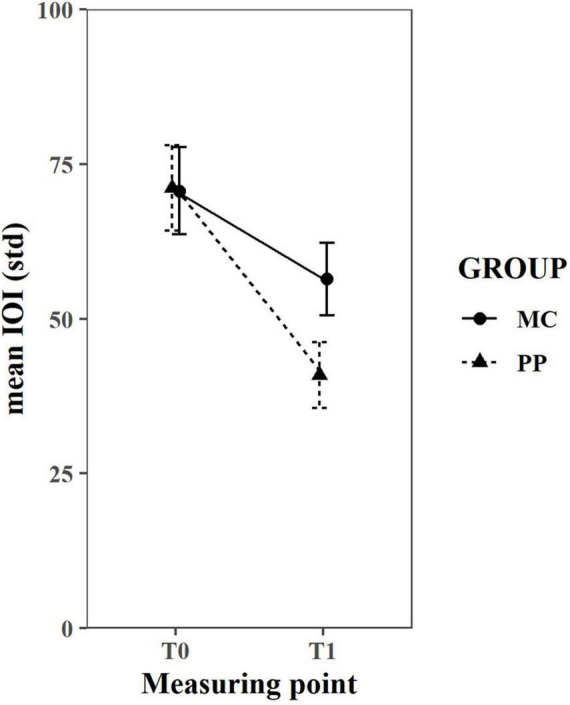
MIDI-based Scale Analysis results. A repeated measures ANOVA revealed a significant time and time*group effect, but no main effect of group. Error bars depict the standard error. T0, baseline measurement; T1, 6 months measurement; IOI, inter-onset interval; MC, music listening/musical culture group; PP, piano practice group, std, standard deviation.

Piano training progress was measures with the MSA. Repeated measures ANOVA revealed a significant time [*F*(1,120) = 44.36, *p* < 0.001] as well as time × group interaction effect [*F*(1,120) = 5.86, *p* = 0.017]. No main effect of group was detected [*F*(1,120) = 1.25, *p* = 0.265]. This means that the PP group improved more in this task than the MC group, which implies a positive training outcome for PP (Figure 5).

## Discussion

To our knowledge, this is the first study investigating how 6 months of musical intervention influences white matter plasticity in a group of initially musically naïve healthy elderly individuals. The main finding is that learning to play the piano, but not participating in music listening/musical culture lessons, stabilized white matter microstructure in the fornix. These microstructural changes appear to be related to piano training intensity. In addition, changes in episodic (long-term) verbal memory performance were positively correlated with microstructural changes across both groups.

### The Fornix and Its Relation to Episodic Memory

The fornix, which is a main output tract of the hippocampus, is part of the limbic system, which is involved in emotional behavior and memory formation (Lövblad et al., 2014). Traditionally, the fornix is associated with episodic memory (Sexton et al., 2010; Metzler-Baddeley et al., 2011) and therefore recollection and not recognition memory (Aggleton and Brown, 2006). While those studies were cross-sectional, the current longitudinal study provides further evidence for these claims, as changes in fornix microstructure positively correlated with episodic (long-term) verbal memory performance. However, this correlation was only small to moderate in size, indicating there are more factors quantifying changes in episodic memory apart from fornix microstructure. This is also shown by a lack of change in episodic (long-term) verbal memory in the MC group over the time of the intervention, although FD decline in the fornix was visible within these 6 months.

While a recent large-scale study in middle-aged and older participants showed that people who engaged with music, including listening, singing, and playing an instrument performed better on an episodic memory task than musically naïve/inactive people (Rouse et al., 2021), it seems plausible that 6 months of lessons is not sufficient to elicit improvements in this cognitive domain.

### PP Intervention Stabilizes Fornix FD

When looking into group differences between the two interventions, the most important finding of this paper is that learning to play the piano seems to stabilize white matter microstructure in the fornix. Although there are many cross-sectional studies showing fornix integrity decreases with age (using FA as a measure; see Douet and Chang, 2015 for a review), the biological underpinnings of these findings are difficult to interpret. The decrease in FA could reflect demyelination, larger axon diameters, lower packing density, increased membrane permeability and/or axons less coherently organized within the voxel (Jones et al., 2013). While FBA simplifies the interpretation of results, it is important to take all three measures in combination into account (Dhollander et al., 2021). In the current study, there was only a significant FD decrease in the MC group, while changes in FC and FDC did not survive Bonferroni correction. This pattern of changes implies either axonal loss or reduced axon diameter with age and not atrophy of the fornix; therefore, there are only microstructural (and not macrostructural) changes. These age-related microstructural changes in the fornix also occurred in other FBA analyses (Adab et al., 2020; Radhakrishnan et al., 2020). Additionally they are supported by a post-mortem study in monkeys that revealed a loss of myelinated nerve fibers and axonal degeneration in the fornix, but no macroscopic changes (Peters et al., 2010). While this fornix degeneration was visible within a time span of 6 months in the MC group, there was no significant change in the PP group.

### PP as a Complex Intervention to Drive Neuroplasticity

When comparing the two interventions to explain the observed beneficial effect of piano lessons, it becomes apparent that learning to play an instrument is a much more complex task than intensive listening. Piano training requires and engages multiple cognitive and sensorimotor processes. Therefore, piano training may counteract naturally occurring age related decline of underlying neural structures (Wan and Schlaug, 2010). Participants in the piano group had to learn and recall specific movements related to musical sounds, coupled to the musical score and therefore specifically engaging the fornix. Especially in the early phases of learning, when one is not quite familiar with reading musical scores, the movement and sound patterns have to be memorized. What might also play a role is that learning to play the piano is a continuous adaptive procedure which involves not only auditory-visual-sensory-motor integration skills but also cognitive abilities throughout learning (Sutcliffe et al., 2020). These are some of the important differences between the two investigated interventions. Therefore, learning a new task may drive cognitive plasticity more strongly (Greenwood and Parasuraman, 2010). Especially when learning to play the piano, corrective feedback not only comes from the teacher during lessons, but also when practicing at home via the auditory and proprioceptive system contributing to improve and perfect performance (Altenmüller, 2003). Therefore, the results simply reflect that continuous training is needed to induce neuroplastic changes. This is supported by the positive correlation of the amount of at home training in the piano group with FD changes in the fornix. In addition, many of the participants spent more time training at home than the required 30 min a day, potentially explained by high intrinsic motivation, which is also recognized as an important factor for neuroplasticity (Sutcliffe et al., 2020). The gradual impact of training intensity on functional and structural brain plasticity, associated with cognitive performance has already been demonstrated in studies matching non-musicians, amateurs, and experts for demographics and musical training onset and intensity (Oechslin et al., 2013, 2018; James et al., 2014).

Interestingly, Burzynska et al. (2017) could show a positive impact of a 6 month dance intervention on fornix integrity in a group of healthy, but low-active elderly participants in comparison to walking and stretching interventions. Dancing is also a complex intervention that shares most of the positive aspects of learning to play an instrument. It is enjoyable, increasingly difficult, requires training with corrective feedback, and is intrinsically rewarding.

### Music-Specific Effects

In the current study, no Bonferroni corrected significant differences between the two interventions were found in the white matter tracts that were previously reported as modified by piano training in the neuroscience of music literature (i.e., the arcuate fasciculus, corticospinal tract and corpus callosum). The most important factor for these differences is probably the age at the start and intensity of musical training. Penhune (2011) suggests that, although neuroplasticity remains possible across the lifespan, there seems to be a sensitive period where musical training has the highest impact on motor learning. Several studies suggest that a start of training in early childhood, preferably before the age of seven, leads to the greatest plastic changes in the corpus callosum (Schlaug et al., 1995; Steele et al., 2013) and also leads to better motor performance (Watanabe et al., 2007). Therefore, it is probable that the sample in this study was too old at the start of training to induce measurable white matter changes in motor related white matter tracts. It is also important to note that the musicians investigated in the above-mentioned studies received intensive training over a long period of time, while the participants analyzed here only trained for 6 months. Yet, that neuroplastic changes induced by musical training are possible and also after shorter periods of training could be demonstrated by Li et al. (2018). In a longitudinal study investigating the effect of piano training in a group of musically naïve young adults (mean age = 23.33 years), they were able to show an increase in FA in a network connecting auditory and sensorimotor regions after six months of training, which also correlated with practice time. This effect receded after another 12 weeks of no training (Li et al., 2018), supporting the hypothesis by Merrett et al. (2013) that continued training is needed to sustain the neuroplastic changes induced by learning a new skill. In sum, there are initial findings in support of the direct effects of musical training on neuroplasticity in the auditory and sensorimotor system, but the current study could not verify this in a sample of older adults.

Further, although it has been suggested that the right arcuate fasciculus is implicated with implicit musical learning (Loui et al., 2011), we could not find any group differences for this tract in our analysis. Although skill acquisition as a type of implicit memory formation is essential for piano playing, we believe that our intervention was too short for our elderly participants and skills were not sufficiently refined after six months. This is supported by studies showing that motor skill learning is strongly age dependent, which is probably also reflected in neuroplastic adaptations (Roig et al., 2014).

### Training and Intervention Studies in People With Cognitive Impairment

Alzheimer’s disease (AD) is the most prevalent form of dementia (Alzheimer’s Association, 2016) and loss of episodic memory is considered one of the first clinical manifestations of AD (Soininen and Scheltens, 1998). There is an increasing interest in the fornix as a potential imaging biomarker for mild cognitive impairment (MCI) and AD, with a variety of studies showing reduced fornix integrity and some even atrophy (see Nowrangi and Rosenberg, 2015 for a review). As medications for AD only show limited benefits, musical interventions have become increasingly popular as a tool to sustain cognitive abilities as long as possible (Leggieri et al., 2019). A recent systematic review and meta-analysis (Dorris et al., 2021) could find a small but significant effect of musical training on cognitive functioning in elderly people with probable MCI or dementia. However, they did not discuss any brain correlates to these cognitive improvements. Cognitive reserve is regarded as one of the main factors that are able to delay age-related cognitive decline or dementia (Stern, 2012). Stern (2012) also suggests cognitive reserve can change over the lifespan and therefore late-life interventions might still be useful. Taking the results of the current study into account, starting to learn to play the piano later in life might be considered an intervention in the challenge of delaying MCI and onset of AD in the elderly by stabilizing white matter microstructure of the fornix.

### Strengths and Limitations

This RCT study comes not only with several strengths but also limitations. For example, having an active control group in this study has many advantages, but it also poses some disadvantages. On the one hand, it allows to draw stronger conclusions for the intervention group and single out the effect of training a new skill. On the other hand, without a passive control group it is not possible to disentangle natural occurring processes from processes related to the active control group. Thus, based on the current data we cannot definitively conclude whether the FD decline in the MC group is natural, slowed down (as compared to community-dwelling elderly) or induced by the activity. However, as there is a vast amount of literature showing a natural decline of fornix microstructure in healthy elderly, and has also been shown by another study within a six months’ time period (Burzynska et al., 2017) we believe the latter unlikely. Still, future studies should also include a passive control group to investigate the specific effect of MC lessons and the slope of natural occurring degeneration of fornix microstructure. Nevertheless, having an intervention study with 155 participants is relatively rare, and to the best of our knowledge, this is the largest study using musical interventions in healthy elderly individuals to date. The use of neuroimaging poses an especially great advantage in the study of apparently healthy elderly individuals, as white matter changes have been shown to precede cognitive deficits in neurodegenerative diseases such as Parkinson’s disease (Rektor et al., 2018) and AD (Caballero et al., 2018). One more limitation is that albeit lesson attendance was monitored to make sure that participants adhered to the study protocol, the homework assessment was based on self-report after six months.

Another limitation of this study is the use of relatively low b-values (b = 1,500 s/mm^2^) during image acquisition. For FBA, it has been recommended to use b-values of 2,000–3,000 s/mm^2^, as the angular contrast increases with higher b-values and therefore extra-axonal water signals are attenuated much stronger (Raffelt et al., 2012, 2015, 2017b; Dhollander et al., 2021). Therefore, although this study had sufficient angular resolution, the relatively low b-values can lead to reduced interpretability of the FD measure as it might not only reflect intra-axonal but also extra-axonal changes (Dhollander et al., 2021). However, as FD decreases and no change in FC in the fornix with age have also been observed in other studies, which used higher b-values (Adab et al., 2020; Radhakrishnan et al., 2020), it is likely that the current interpretation remains valid. Future studies should still aim to use higher b-values in their scanning protocols to improve the interpretability of results.

## Conclusion

In conclusion, learning to play the piano seems to be a promising activity to stabilize white matter microstructure in the fornix, as it is adaptive, enjoyable and intrinsically rewarding. The current results show that, while 6 months of piano training might not be able to reverse microstructural changes in the fornix, it is still important to start interventions before the onset of any clinical symptoms, as degeneration was visible in the active control group even within this relatively short time period. Future studies are needed to investigate whether continued training is needed to maintain this effect and whether it could be a suitable candidate to prevent or delay neurodegenerative diseases affecting the fornix, such as AD.

## Data Availability Statement

The raw data supporting the conclusions of this article will be made available by the authors, without undue reservation.

## Ethics Statement

The studies involving human participants were reviewed and approved by the Research Ethics Review Committee of Leibniz University Hanover and the Ethics Committee of Hannover Medical School (number 3604-2017) as well as the Cantonal Ethics Committee Geneva (number 2016-02224). The patients/participants provided their written informed consent to participate in this study.

## Author Contributions

KJ: conceptualization, investigation, data curation, formal analysis, and writing—original draft preparation. DM: conceptualization, investigation, preprocessing of T1 Data, and writing—review and editing. FW: conceptualization, investigation, and writing—review and editing. DS, FG, and DV: conceptualization and writing—review and editing. MK: detailed input on funding acquisition, conceptualization, and writing—review and editing. CJ: funding acquisition, conceptualization, and writing—review and editing. EA: funding acquisition, conceptualization, supervision, and writing—review and editing. TK: detailed input on funding acquisition, conceptualization, supervision, and writing—review and editing. CS: conceptualization, supervision, and writing—review and editing. All authors contributed to the article and approved the submitted version.

## Conflict of Interest

The authors declare that the research was conducted in the absence of any commercial or financial relationships that could be construed as a potential conflict of interest.

## Publisher’s Note

All claims expressed in this article are solely those of the authors and do not necessarily represent those of their affiliated organizations, or those of the publisher, the editors and the reviewers. Any product that may be evaluated in this article, or claim that may be made by its manufacturer, is not guaranteed or endorsed by the publisher.

## Footnotes

1
https://3Tissue.github.io
